# Real-World Use of Intradetrusor Botulinum Toxin Injections: A Population-Based Study from France

**DOI:** 10.3390/toxins16100423

**Published:** 2024-10-01

**Authors:** Alain Ruffion, Pierre Karam, Anne Forestier, Pierre Denys

**Affiliations:** 1 Department of Urology, Hospices Civils de Lyon, Lyon Sud Hospital, 69495 Pierre-Bénite, France; 2 PKCS, 69130 Ecully, France; p.karam@pkcs.fr; 3 Ipsen, 92100 Boulogne-Billancourt, France; anne.forestier@ipsen.com; 4 Neuro-Uro-Andrology Department, Raymond Poincaré Hospital, 92380 Garches, France; pierre.denys@aphp.fr

**Keywords:** botulinum toxin, intradetrusor, care pathway, neurological disorders, overactive bladder

## Abstract

In recent decades, intradetrusor injections of botulinum toxin A (BoNT-A) have been widely applied to treat incontinence in both idiopathic overactive bladder (iOAB) and neurogenic detrusor overactivity incontinence (NDOI). This analysis, based on the French National Hospital Discharge Database (PMSI), aims to describe real-world trends in intradetrusor BoNT-A use between 2014 and 2022. Among 32,864 patients who received at least one intradetrusor BoNT-A injection, 18,320 (55.7%) had conditions coded under iOAB, 13,376 (40.7%) under NDOI, and 1168 (3.6%) under other indications. The overall mean interval between two intradetrusor BoNT-A injections was 9.7 months, ranging from 8.7 months in patients with multiple sclerosis (MS) to 11.5 months in patients with cerebral pathologies. The median number of injections was two (quartile 1–quartile 3, 1–4) in patients with spina bifida, whereas it was five (2–10) in those with MS. Only 31% of patients with iOAB received more than two intradetrusor BoNT-A injections. Regardless of its indication, BoNT-A was well tolerated. Adverse events occurring during or requiring hospitalization included infections (3.8%), hematuria (0.53%), and bleeding episodes necessitating transfusions (0.11%), all recorded within the initial month following BoNT-A injection. Our analysis of the PMSI database highlights a broad spectrum of intradetrusor BoNT-A injection practices.

## 1. Introduction

Overactive bladder (OAB) is a common storage syndrome marked by urinary urgency (with or without urgency incontinence), urinary frequency, and nocturia [1,2]. OAB can significantly impact quality of life, affecting emotional, social, sexual, occupational, and physical aspects of daily life [1,2]. OAB can have a neurogenic or idiopathic origin (iOAB) [3].

Anticholinergic agents are commonly considered the first-line pharmacological treatment for OAB [1,2]. When initial conservative measures and anticholinergic agents prove to be ineffective or are poorly tolerated, the use of intradetrusor injections of botulinum toxin A (BoNT-A) is recommended as a second-line treatment of OAB [1,2,4]. BoNT-A is a potent neurotoxin that acts by blocking the release of acetylcholine and neurotransmitters that are soluble N-ethylmaleimide-sensitive factor attachment protein receptor (SNARE)-dependent at the presynaptic neuromuscular junction [1,2,4,5,6]. BoNT-A may also modulate the expression or activity of suburothelial sensory receptors. The combined effect of blocking neurotransmitter release and modulating sensory receptors causes denervation of the detrusor muscle [1,2,4,5,6].

Repeated injections of BoNT-A into the detrusor muscle have been shown to be safe and effective in patients with OAB, leading to reduced maximal detrusor pressure, increased bladder capacity, and increased reflex volume, with no negative effects on bladder compliance and no evidence of increased drug resistance [1,2,4,5,6,7,8,9]. When injected into the detrusor muscle, BoNT-A typically provides therapeutic effects for 6 to 9 months, and effects may sometimes last up to 12 months [1,5,6,7,8]. 

Neurological disorders such as multiple sclerosis (MS), spina bifida, and spinal cord injury (SCI) can modify lower urinary tract function and lead to neurogenic detrusor overactivity incontinence (NDOI), which can manifest as OAB [10]. These specific etiologies were the focus of the initial clinical studies that subsequently led to the reimbursement of BoNT-A (starting in August 2012 in France). Subsequently, due to the higher prevalence of iOAB [3] and the notable efficacy of BoNT-A in this condition [11], later clinical studies paved the way for the possible reimbursement and use of BoNT-A in iOAB as well (beginning in May 2014) [12]. 

Despite the positive effects of intradetrusor BoNT-A injections in patients with OAB, there is a dearth of real-world data on its use in this patient population. Accordingly, the current study, based on an analysis of the French National Hospital Discharge Database (*Programme de Médicalisation des Systèmes d’Information*, PMSI), aims to describe the trends in intradetrusor BoNT-A use between 2014 and 2022. In France, intradetrusor BoNT-A injections are only permitted during hospitalization, underscoring the relevance of such an analysis.

## 2. Results

### 2.1. Overall Trends in Intradetrusor BoNT-A Use

**Figure 1:**
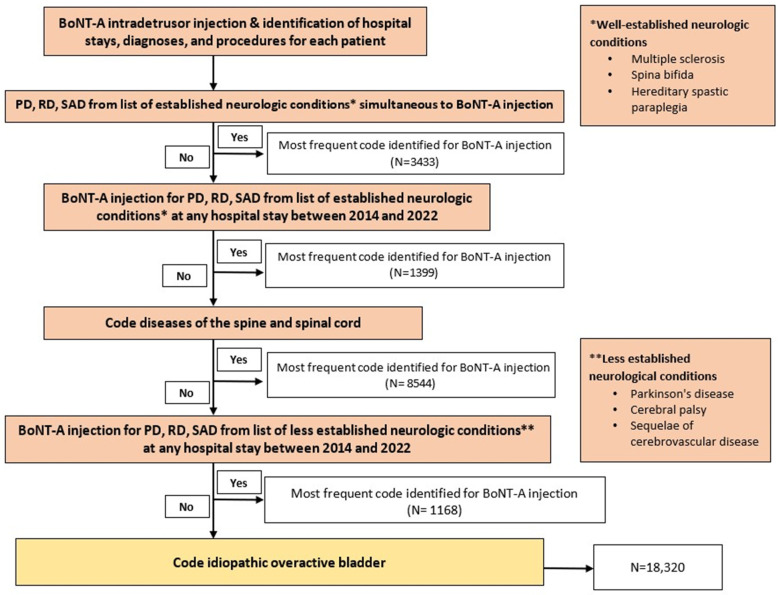
Process of selection of patients by reason for botulinum toxin type A (BoNT-A) injection. Abbreviations: PD, primary diagnosis; RD, related diagnosis; SAD, significantly associated diagnosis. N = most frequent code identified for BoNT-A injection. * refers to well-established neurologic conditions, including multiple sclerosis, spina bifida, and hereditary spastic paraplegia. ** refers to less established neurologic conditions, including Parkinson’s disease, cerebral palsy, and sequelae of cerebrovascular disease.

Between 2014 and 2022, 32,864 patients received at least one intradetrusor BoNT-A injection, with 18,320 (55.7%) coded with iOAB, 13,376 (40.7%) with NDOI, and 1168 (3.6%) as less established NDOI (Figure 1). The PMSI database recorded a total of 98,483 intradetrusor BoNT-A injections during the same period. The vast majority of intradetrusor BoNT-A injections (over 90%) were performed in an outpatient setting. All 32,864 evaluated patients (100%) had a repeat BoNT-A injection. The median (quartile 1–quartile 3) interval between two BoNT-A injections was 210 (182–331) days, and the mean ± standard deviation (SD) interval was 295 ± 241 days. Most repeated BoNT-A injections (56.0%) were administered between 180 and 365 days, whereas 22.3% and 15.1% of repeated BoNT-A injections occurred between 90 and 180 days and between 365 and 730 days, respectively.

**Figure 2:**
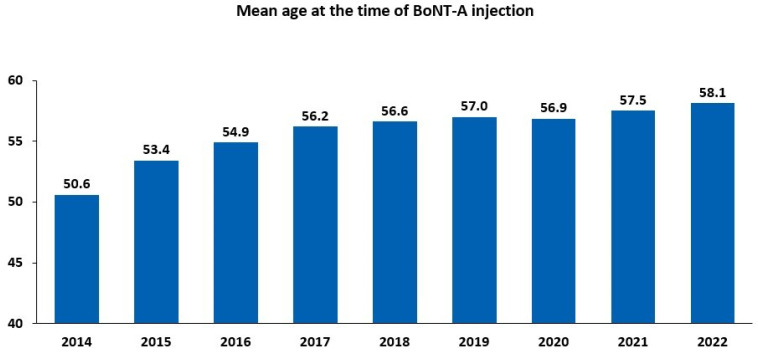
Intradetrusor use of botulinum toxin type A (BoNT-A) between 2014 and 2022 according to age.

There has been a steady increase in both the number of BoNT-A injections and the count of treated patients since 2014 (Figure S1). Between 2014 and 2022, the number of administered injections increased by 165.7%, and the number of treated patients increased by 184.2%. The increase in the number of BoNT-A-treated patients between 2014 and 2022 was reflected by a rise in the mean age of the patients, ranging from 50.6 years in 2014 to 58.1 years in 2022 (Figure 2). Between 2014 and 2022, women were more likely to be treated than men, accounting for almost two-thirds (9971/15,297; 65.2%) of intradetrusor BoNT-A injections in 2022 (Table 1).

### 2.2. Indications for the Administration of Intradetrusor BoNT-A Injections

**Figure 3:**
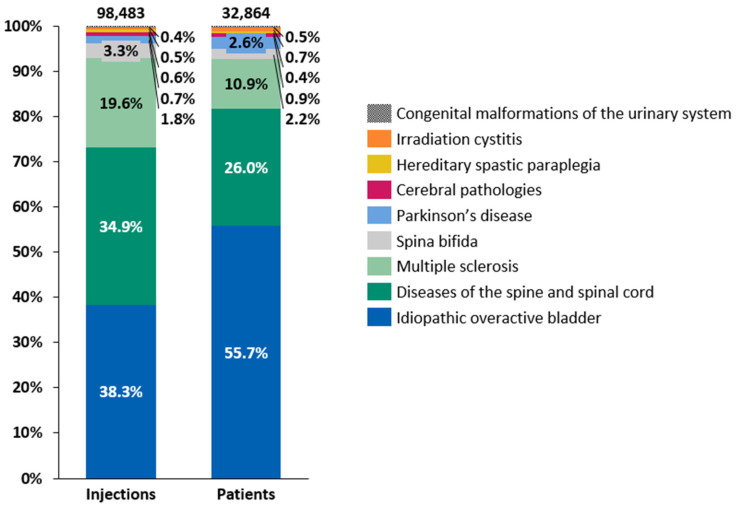
Distributions of patients and intradetrusor injections of botulinum toxin type A by etiology between 2014 and 2022.

Intradetrusor BoNT-A injections were administered for various conditions between 2014 and 2022, with the distribution of patients and the number of injections by etiology following a consistent pattern throughout the period of 2014–2022 (Figure 3). iOAB accounted for 38.3% of all injections and 55.7% of all treated patients, while diseases of the spine and spinal cord represented 34.9% of all injections and 26.0% of all treated patients. MS contributed to 19.6% of all injections and 10.9% of all treated patients. Although the number of administered intradetrusor BoNT-A injections increased consistently between 2014 and 2022 across various etiologies, the growth was less pronounced between 2019 and 2022 than in previous years (Table 2). Specifically, the growth in intradetrusor BoNT-A injections between 2019 and 2022 averaged 5.6% per year. The etiology with the highest injection increase between 2014 and 2022 was iOAB, rising from 1227 injections in 2014 to 6516 in 2022, accounting for a 431% increase. This was followed by Parkinson’s disease, albeit a relatively small group, which rose from 73 intradetrusor BoNT-A injections in 2014 to 259 in 2022, showing a 255% increase. Conversely, spina bifida had one of lowest increases, with a 23% rise from 372 injections in 2014 to 459 in 2022. Across different etiologies, the mean interval between two intradetrusor BoNT-A injections ranged from 264.5 days in patients with MS to 351.2 days in patients with cerebral pathologies (i.e., cerebral palsy and sequelae of cerebrovascular disease) (Figure S2).

**Figure 4:**
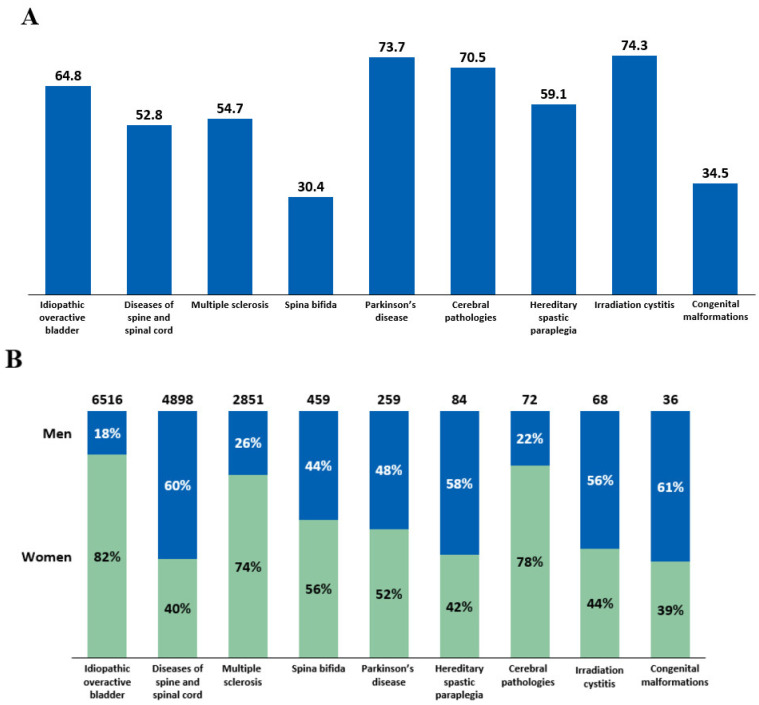
(**A**) Mean age of patients receiving intradetrusor botulinum toxin type A (BoNT-A) injections in 2022 according to etiology. (**B**) BoNT-A treatment rates in men and women in 2022 categorized by etiology.

Intradetrusor BoNT-A treatment rates in 2022 were analyzed by age and sex across different etiologies (Figure 4). The mean age of patients who received intradetrusor BoNT-A injections varied, ranging from 30.4 years in patients with spina bifida to 74.3 years in those with irradiation cystitis (Figure 4A). The male-to-female BoNT-A treatment ratios also varied depending on the etiology (Figure 4B). Notably, there was a female predominance in intradetrusor BoNT-A use in iOAB (82%), cerebral pathologies (78%), and MS (74%), whereas male predominance was noted in diseases of the spine and spinal cord (60%). Specifically, in spina bifida, 44% of patients treated with BoNT-A were men, while 56% were women.

**Figure 5:**
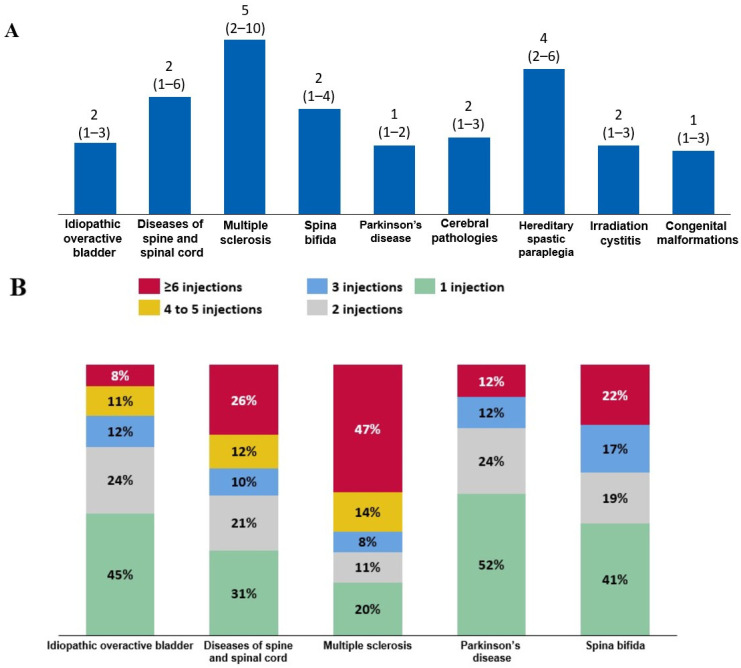
(**A**) Median (quartile 1–quartile 3) number of injections between 2016 and 2022 among patients who initiated intradetrusor botulinum toxin type A (BoNT-A) therapy in 2016. (**B**) Frequency of BoNT-A injections among these patients according to etiology.

When the trends in BoNT-A use were evaluated in patients who initiated BoNT-A therapy in 2016 and were followed-up until 2022, the median (quartile 1–quartile 3) number of intradetrusor BoNT-A injections was two (1–4) in patients with spina bifida, whereas it was five (2–10) in those with MS. Patients with Parkinson’s disease were at the lower end of the range, receiving a median of one (1–2) injection (Figure 5A). Consistently, among all patients who initiated BoNT-A therapy in 2016, 47% of patients with MS received at least six BoNT-A injections (Figure 5B).

### 2.3. Safety of Intradetrusor BoNT-A

Intradetrusor BoNT-A injections were well tolerated overall. Among a total of 96,800 intradetrusor BoNT-A injections administered during hospital stays between 2014 and 2022, there were 3681 recorded infections (3.8%) within the first month of the BoNT-A injection. In addition, 510 cases of hematuria (0.53%) were recorded within the first month, including 226 on the day of injection and 227 cases 1 to 7 days post injection. Furthermore, 104 cases of bleeding episodes requiring transfusions (0.11%) were reported, including 12 on the day of the injection and 30 transfusions 1 to 7 days post injection.

The rate of reported hematuria was significantly higher in patients receiving intradetrusor BoNT-A for NDOI and less established NDOI than in those being treated for iOAB (0.51% versus 0.32%; *p* < 0.001). Following intradetrusor BoNT-A injection, there was a notable increase in the rate of hospitalization for infection. Among patients with iOAB, the rate of hospitalization for infection increased from 0.08% in the month before injection to 0.20% in the month after injection (*p* < 0.001). Similarly, among patients with NDOI and less established NDOI, the rate increased from 0.21% to 0.37% in the same time frame (*p* < 0.001). The rate of hospitalization for infections in the medium term showed no significant from before to after BoNT-A injection, with overall rates of 1.88% before and 1.84% after (*p* = 0.72).

## 3. Discussion

In this 9-year longitudinal analysis of the French PMSI database, we observed a diverse range of intradetrusor BoNT-A injection practices among a total of 32,864 patients treated with BoNT-A, with 55.7% receiving intradetrusor BoNT-A for iOAB, 40.7% for NDOI, and 3.6% for less established NDOI. While accumulating evidence supports the clinical and urodynamic benefits of intradetrusor BoNT-A therapy, along with its safety profile, it is noteworthy that available data stem from small-scale clinical studies focused on patients with NDOI caused by SCI or MS [8,9,10,13,14,15,16,17].

Nevertheless, in recent decades, BoNT-A has been widely applied to treat lower-urinary-tract dysfunctions, particularly in cases that are difficult to manage with oral pharmacologic medications [14]. The wide applicability of intradetrusor BoNT-A therapy is underscored by our study, revealing a significant 165.7% increase in the number of BoNT-A-injections between 2014 and 2022. Specifically, the growth in intradetrusor BoNT-A injections between 2019 and 2022 averaged 5.6% per year. According to an epidemiologic review of OAB causes [15], the rise in BoNT-A use reflects not only an increase in the prevalence of OAB—both idiopathic and neurogenic—but also an expanding acceptance of BoNT-A as an effective treatment option. Indeed, despite the COVID-19 pandemic (2020–2022), which led to a reduction in treatments for many patients with non-urgent urologic conditions in France, the number of intradetrusor BoNT-A injections continued to rise in our study. This highlights the value of BoNT-A treatment for both patients and practitioners, as well as its convenience and ease of use, since it is predominantly administered in outpatient settings under local anesthesia. 

Our study further revealed that the mean interval between two intradetrusor BoNT-A injections was 295 days or 9.7 months. The reinjection interval varied between 8.7 months in patients with MS and 11.5 months in patients with cerebral pathologies. This observation aligns with findings from several clinical trials, reporting an average reinjection interval spanning 8 to 11 months [5,8,9,16,17]. This highlights the consistent use of intradetrusor BoNT-A treatment across diverse neurological and urological conditions. This consistency also reflects the validity of our algorithms in identifying underlying diseases based on the PMSI database. 

As anticipated, the median number of BoNT-A injections varied considerably by etiology, reaching as high as five in patients with MS but remaining lower than three for other BoNT-A indications. Notably, patients with Parkinson’s disease received a median of one injection compared to a median of five in patients with MS. Although the exact reasons for this lower median are unclear, possible explanations may include the reduced efficacy of BoNT-A, the severity of the underlying condition, patient compliance, risks associated with age-related retention issues including prostate problems in men, and catheterization challenges. Indeed, bladder dysfunction in Parkinson’s disease is variable and multifactorial, involving not only neurogenic detrusor overactivity but also other factors, such as bladder outlet obstruction, detrusor underactivity, detrusor–sphincter dyssynergia, or impaired bladder sensation [18,19]. Consequently, treatment approaches may differ based on these factors and the perceived effectiveness of intradetrusor BoNT-A injections [19]. Similar trends of lower injection numbers were also observed in patients treated for irradiation cystitis, iOAB, and cerebral pathologies. For iOAB, despite a significant increase in the use of intradetrusor BoNT-A injections between 2014 and 2022, only 31% of our cohort received more than two injections. A potential limitation in our study is the duration of the effectiveness of BoNT-A in this specific indication, which may have contributed to the lower number of injections. Nevertheless, the length of the study period should mitigate this effect.

Another long-term retrospective analysis from France performed between 2005 and 2012 revealed a low rate of persistence of intradetrusor BoNT-A therapy administered for OAB [17]. The median number of BoNT-A injections per patient was two, with the main causes of BoNT-A discontinuation being lack of efficacy (36%), persistent improvement of symptoms not requiring any reinjections (24%), and tolerability issues (20%). It is worth noting that male sex was significantly associated with intradetrusor BoNT-A therapy discontinuation [17]. This finding is reflected in our analysis, in which, overall, women accounted for almost two-thirds of intradetrusor BoNT-A injections in 2022. To enhance the use of intradetrusor BoNT-A treatment for iOAB and NDOI, it has been proposed that patients should not receive repeated injections if they experience ongoing compliance issues, no or minimal urodynamic or symptomatic improvement after two injections, or severe adverse events (AEs) [16]. Regular evaluation of urodynamic parameters, including maximum detrusor pressure, bladder compliance, bladder capacity, and non-voiding contractions, also remains crucial for the individualization of intradetrusor BoNT-A therapy [20]. 

In this real-world heterogeneous patient population, BoNT-A was well tolerated, with all AEs occurring at a rate of less than 5%. This aligns with consistent findings from clinical studies that have also reported a favorable safety profile for intradetrusor BoNT-A therapy, with a minimal risk of systemic side effects. The most commonly reported adverse reactions for intradetrusor BoNT-A include urinary tract infections (UTIs), urinary retention, and mild hematuria [8,9,16,21,22]. Importantly, in our analysis, causality between intradetrusor BoNT-A therapy and AEs could not be firmly established due to the retrospective observational nature of the study and the potential for confounding factors. Additionally, transfusions were not reported in previous BoNT-A studies for NDO [8,9,16,21,22]. Most AEs reported in these studies were mild, making it difficult to link intradetrusor BoNT-A injections to serious AEs like bleeding episodes that require transfusions. Nonetheless, it is advisable that healthcare practitioners adequately inform patients about the potential risks of BoNT-A-related AEs before initiating intradetrusor BoNT-A injections [16]. The identification of predictors of AEs could also aid in counselling of patients initiating intradetrusor BoNT-A therapy [23]. Previous UTIs, an in situ prostate in males, and clean intermittent self-catheterization have been associated with a higher incidence of UTIs after BoNT-A therapy [14,23].

This study has some limitations due to its reliance on retrospective electronic health record data. These include potential biases, missing data, and coding errors, given that the coding was performed by treating physicians and depends entirely on their accuracy in selecting medical codes. Additionally, we did not have access to data on the type of BoNT-A preparation used or the administered dosage. Furthermore, the PMSI database does not capture concomitant medications or outpatient treatments and diagnoses managed in community settings, including complications such as UTIs treated outside of the hospital. Nevertheless, our analysis is strengthened by the long observation period, with comprehensive data collection on intradetrusor BoNT-A use. Our study is also strengthened by the use of the PMSI national database, which covers the entire population of France (over 66 million people), minimizing the risk of selection bias. To the best of our knowledge, this study is the first to utilize a national database to investigate real-life intradetrusor BoNT-A treatment practices.

## 4. Conclusions

Since 2014, the use of intradetrusor BoNT-A therapy has been steadily increasing in France for both iOAB and NDOI. Among 32,864 patients treated with BoNT-A between 2014 and 2022, a broad spectrum of intradetrusor BoNT-A injection practices was observed. The median number of injections varied widely, ranging from one in patients with Parkinson’s disease to two in those with iOAB and five in those with MS. Importantly, regardless of its indication, intradetrusor BoNT-A therapy demonstrated excellent tolerability. As the landscape of neurological conditions treated with intradetrusor BoNT-A continues to evolve, caution is advised, and ongoing research is warranted to refine guidelines and enhance patient care.

## 5. Materials and Methods

### 5.1. Study Design and Data Source

This was a nationwide, population-based, retrospective cohort study performed between 2014 and 2022. Data were obtained from the PMSI database, which is a comprehensive database that includes standardized discharge summaries from all inpatients and outpatients in public hospitals, as well as inpatients from private hospitals, in France, covering a total population of over 66 million individuals [13]. 

Each PMSI record includes the following details: administrative details (i.e., age, gender, residence code, and the unique identifier of each patient), information on the medical procedures conducted during hospitalization, and diagnostic information. Diagnoses are coded according to the International Classification of Diseases version 10 (ICD-10), categorized as the primary diagnosis (the reason behind the patient’s hospitalization), related diagnoses (all conditions potentially related to the primary diagnosis), or significantly associated diagnoses (all complications and morbidities that could influence the course of the hospitalization). Medical procedures are coded according to the French Joint Classification of Medical Procedures (*Classification Commune des Actes Médicaux*, CCAM).

This study was performed in accordance with French laws and regulations and the Declaration of Helsinki and was reported to the French Data Protection Agency (*Commission Nationale de l’Informatique et des Libertés*, CNIL). Under French law and CNIL guidelines, formal ethical approval and informed consent were not required, as this study did not involve the direct participation of individuals and all extracted data were anonymized.

### 5.2. Study Population

We first queried the PMSI database to identify all patients who received at least one intradetrusor BoNT-A injection between 2014 and 2022. CCAM codes JDLE900 and JDLE332 were used to identify patients who received an intradetrusor injection of BoNT-A via cystoscopy. Both of these codes refer to the “injection of botulinum toxin into the bladder muscle via urethrocystoscopy”. It is worth noting that JDLE900 was removed from the CCAM codes in 2014 and was only used to identify patients treated with BoNT-A in the year 2014. The implementation of such a change may have taken longer in some institutions. For each patient who received at least one intradetrusor BoNT-A injection, we collected data on all inpatient diagnoses, inpatient and outpatient stays, and performed medical procedures. We identified the conditions for which intradetrusor BoNT-A may have been indicated using ICD-10 codes from primary, related, or significantly associated diagnoses (Table 3). ICD-10 codes are part of the International Classification of Diseases, 10th Edition, a standardized coding system used globally to classify and code diseases and symptoms [24].

Figure 1 presents a flow chart illustrating the patient selection process based on the reason for BoNT-A injection. Our approach involved a step-by-step verification, starting from the most certain diagnosis to minimize data loss and data duplication. Initially, we classified patients who received at least one coded intradetrusor BoNT-A injection and had a simultaneously coded disease such as MS, spina bifida, or hereditary spastic paraplegia. We then applied another criterion to the remaining population, selecting those with a disease code from any hospital stay between 2014 and 2022 that differed from the one associated with the BoNT-A injection. In cases where BoNT-A was administered for multiple conditions in a single patient, we retained only the condition that was most frequently coded. Consequently, each patient had only one condition recorded that justified BoNT-A administration in our analysis.

Overall, this selection process resulted in the categorization of conditions into the following three groups based on their neurological origin: well-established neurological conditions leading to a neurogenic bladder because of spinal cord dysfunction (including MS, spina bifida, hereditary spastic paraplegia, and diseases of the spine and spinal cord), established non-neurological conditions/symptoms encompassing iOAB, and less established conditions that may have neurological involvement (including Parkinson’s disease, cerebral palsy, and sequelae of cerebrovascular disease). By categorizing conditions based on their neurological origin (i.e., iOAB, NDOI, and less established NDOI), this approach allows for the identification of specific trends and preferences in intradetrusor BoNT-A use across different patient populations. We were also able to distinguish “tentative indications” such as radiation-induced cystitis or interstitial cystitis.

### 5.3. Outcomes

Multiple outcomes were evaluated among hospitalized patients, such as the number of patients who underwent at least one intradetrusor BoNT-A injection between 2014 and 2022 and the percentage change, the number of intradetrusor BoNT-A injections administered between 2014 and 2022 and the percentage change, and the time interval between two BoNT-A injections. Furthermore, all patients treated with BoNT-A were analyzed according to the injection indication, age, and sex. We also evaluated the safety profile of intradetrusor BoNT-A injections, particularly their risks of infection (excluding cystitis and assessed using ICD-10 codes N10, N41.0, N45.0, N45.9, A40, A41, and R572), hematuria (ICD-10 code R31), and transfusion (assessed using CCAM codes FELF004, FELF011, and FELF001) recorded during hospitalization within 30 days after BoNT-A injection. In addition, to estimate whether an injection corresponded to the initiation of BoNT-A therapy, we applied a 2-year washout period (i.e., 2014 and 2015) and began the analysis with patients who received an injection in 2016, following them up until 2022.

### 5.4. Statistical Analysis

Descriptive analyses were conducted using frequency and percentage for categorical variables and mean values with their SD or median values with first and third quartiles for continuous variables. Statistical comparisons between groups were performed using the chi-square test. No imputation of missing data was performed, and all coding was discontinued in cases of patient death. All analyses were performed using SQL server software (version 2022; Microsoft, Redmond, WA, USA).

## Figures and Tables

**Table 1:** Intradetrusor use of botulinum toxin type A between 2014 and 2022 according to sex.

Year		2014	2015	2016	2017	2018	2019	2020	2021	2022
**Men**	**n (%)**	2567 (44.5)	2982 (39.4)	3363 (37.2)	3851 (36.2)	4183 (35.7)	4523 (34.8)	4292 (35.5)	4891 (34.0)	5326 (34.8)
**Women**	**n (%)**	3201 (55.5)	4579 (60.6)	5681 (62.8)	6773 (63.8)	7521 (64.3)	8486 (65.2)	7802 (64.5)	9497 (66.0)	9971 (65.2)
**Total number of patients**		5768	7561	9044	10,624	11,704	13,009	12,094	14,388	15,297

**Table 2:** Increase in the number of intradetrusor botulinum toxin type A injections by etiology between 2014 and 2022.

Condition	2014	2015	2016	2017	2018	2019	2020	2021	2022	Percentage Change between 2014 and 2020
**Congenital malformations of urinary system**	30	33 (+10%)	33 (0%)	30 (−9%)	50 (+67%)	44 (−12%)	37 (−16%)	59 (+59%)	36 (−39%)	+20%
**Irradiation cystitis**	20	40 (+100%)	66 (+65%)	71 (+8%)	69 (−3%)	68 (−1%)	58 (−15%)	63 (+9%)	68 (+8%)	+240%
**Hereditary spastic paraplegia**	54	49 (−9%)	53 (+8%)	50 (−6%)	58 (+16%)	71 (+22%)	70 (−1%)	76 (+9%)	84 (+11%)	+56%
**Cerebral pathology**	40	52 (+30%)	76 (+46%)	97 (+28%)	85 (−12%)	87 (+2%)	74 (−15%)	91 (+23%)	72 (−21%)	+80%
**Parkinson’s disease**	73	113 (+55%)	156 (+38%)	205 (+31%)	252 (+23%)	270 (+7%)	206 (−24%)	236 (+15%)	259 (+10%)	+255%
**Spina bifida**	372	292 (−22%)	310 (+6%)	304 (−2%)	324 (+7%)	381 (+18%)	336 (−12%)	438 (+30%)	459 (+5%)	+23%
**Multiple sclerosis**	1303	1589 (+22%)	1813 (+14%)	2094 (+16%)	2259 (+8%)	2573 (+14%)	2100 (−18%)	2718 (+29%)	2851 (+5%)	+119%
**Diseases of the spine and spinal cord**	2617	2968 (+13%)	3314 (+12%)	3790 (+14%)	3980 (+5%)	4265 (+7%)	3928 (−8%)	4594 (+17%)	4898 (+7%)	+87%
**Idiopathic overactive bladder**	1227	2384 (+94%)	3192 (+34%)	3941 (+23%)	4579 (+16%)	5171 (+13%)	4667 (−10%)	6052 (+30%)	6516 (+8%)	+431%
**Total**	5736	7520 (+31%)	9013 (+20%)	10,582 (+17%)	11,656 (+10%)	12,930 (+11%)	11,476 (−11%)	14,327 (+25%)	15,243 (+6%)	+166%

**Table 3:** Conditions for which intradetrusor botulinum toxin type A might be indicated.

Condition		ICD-10 Code
***List of main etiologies***		
Multiple sclerosis		G35
Spina bifida		Q05
Other congenital malformations of the urinary system		Q64
Hereditary spastic paraplegia		G11.4
Irradiation cystitis		N30.4
Diseases of the spine and spinal cord	Other and unspecified diseases of the spinal cord	G95
	Sequelae of injuries of the neck and trunk	T91
	Injury of nerves and the spinal cord at the neck level	S14
	Injury of nerves and the spinal cord at the thorax level	S24
	Injury of the lumbar and sacral spinal cord and nerves at the abdomen, lower back, and pelvis levels	S34
	Nerve-root and plexus compressions in diseases classified elsewhere	G55
	Spinal stenosis	M480
	Hemiplegia and hemiparesis	G81
	Paraplegia and quadriplegia	G82
	Other paralytic syndromes	G83
***Symptoms or less defined etiologies***		
Parkinson’s disease		G20
Cerebral palsy		G80
Sequelae of cerebrovascular disease		I69
Idiopathic overactive bladder	Uninhibited neuropathic bladder not classified elsewhere	N31.0
	Reflex neuropathic bladder not classified elsewhere	N31.1
	Flaccid neuropathic bladder not classified elsewhere	N31.2
	Other neuromuscular dysfunctions of the bladder	N31.8
	Unspecified neuromuscular dysfunction of the bladder	N31.9
	Unspecified urinary incontinence	R32
	Stress incontinence	N39.3
	Other specified urinary incontinence	N39.4

## Data Availability

Restrictions apply to the availability of these data, since the data underlying this publication were provided by the *Agence Technique de l’Information sur l’Hospitalisation* (ATIH) under contract to Ipsen.

## Supplementary Materials

The following supporting information can be downloaded at: https://www.mdpi.com/article/10.3390/toxins16100423/s1, Figure S1: Changes in the number of patients who received at least one intradetrusor botulinum toxin type A (BoNT-A) injection and in the number of intradetrusor BoNT-A injections between 2014 and 2022; Figure S2: Mean interval (days) between two botulinum toxin type A (BoNT-A) injections according to etiology.
