# Supplementary material for: Real-World Use of Intradetrusor Botulinum Toxin Injections: A Population-Based Study from France
Source: Toxins (Basel). 2024 Oct 1;16(10):423. doi: 10.3390/toxins16100423 (PMC11511096; doi:10.3390/toxins16100423)
Supplement: Supplementary file 1 [file toxins-16-00423-s001.zip › toxins-3168430-supplementary.pdf]

Supplementary Materials: Real-world use of intradetrusor botulinum toxin injections: A population-based study from France

Alain Ruffion,, Pierre Karam, Anne Forestier and Pierre Denys

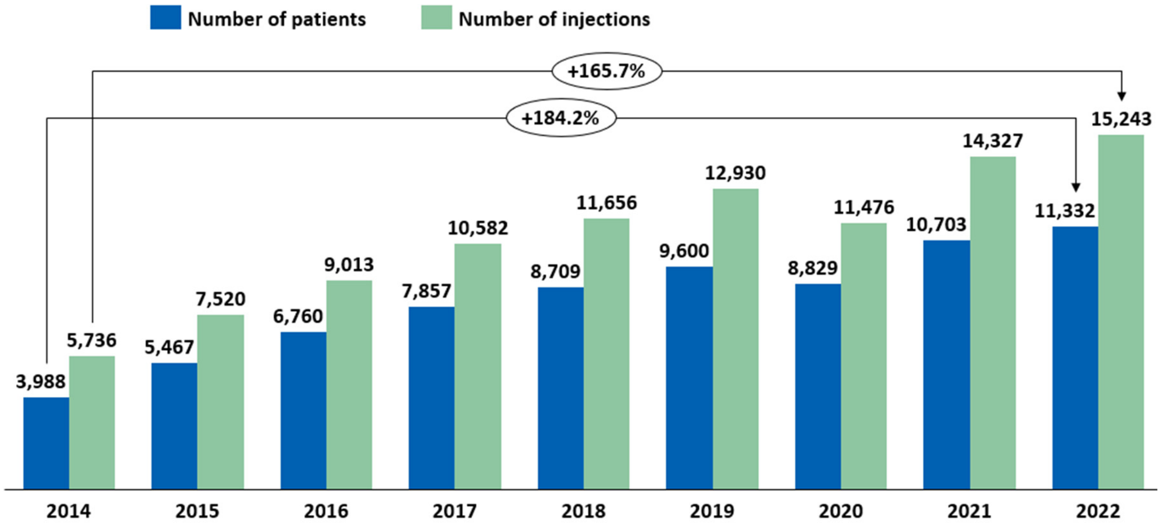

Figure S1. Changes in the number of patients who received at least one intradetrusor botulinum toxin type A (BoNT-A) injections and in the number of intradetrusor BoNT-A injections between 2014 and 2022

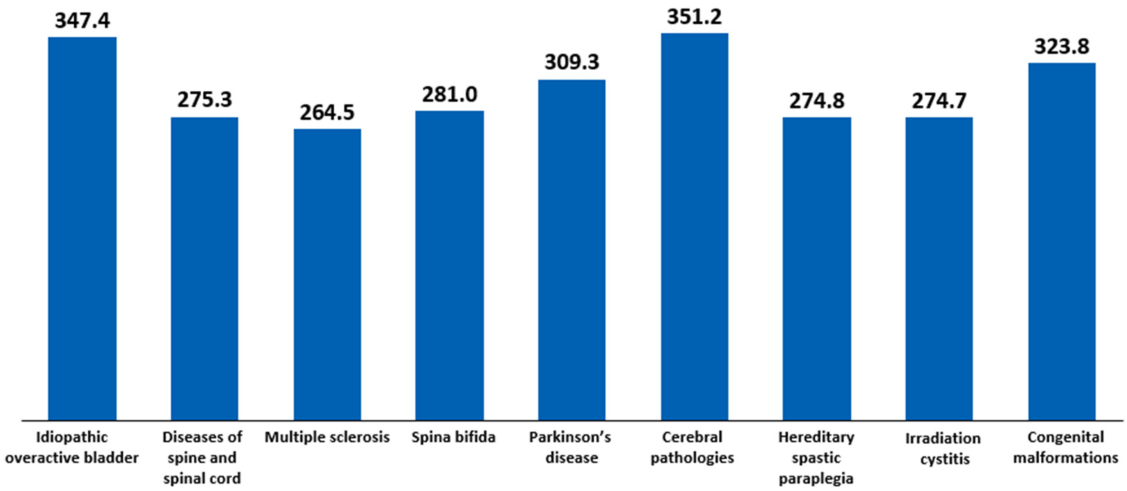

Figure S2

Figure S2. Mean interval (days) between two botulinum toxin type A (BoNT-A) injections according to etiology.
